# Supplementary material for: Impact of Early Intrapatient Variability of Tacrolimus Concentrations on the Risk of Graft-Versus-Host Disease after Allogeneic Stem Cell Transplantation Using High-Dose Post-Transplant Cyclophosphamide
Source: Pharmaceuticals (Basel). 2022 Dec 9;15(12):1529. doi: 10.3390/ph15121529 (PMC9784628; doi:10.3390/ph15121529)
Supplement: Supplementary file 1 [file pharmaceuticals-15-01529-s001.zip › pharmaceuticals-2033039-supplementary.pdf]

Impact of early intrapatient variability of tacrolimus concentrations on the risk of graft-versus-host disease after allogeneic stem cell transplantation using high-dose post-transplant cyclophosphamide

Daniel N. Marco [1], María Queralt Salas [1], Gonzalo Gutiérrez-García [1], Inés Monge [2], Gisela Riu [2], Esther Carcelero [2], Joan Ramón Roma [2], Noemí Llobet [1], Jordi Arcarons [1], María Suárez-Lledó [1], Nuria Martínez [1], Alexandra Pedraza [1], Ariadna Domenech [1], Laura Rosiñol [1], Francesc Fernández-Avilés [1], Álvaro Urbano-Ispízua [1], Montserrat Rovira [1], Mercè Brunet [3] and Carmen Martínez [1]

[1] Hematopoietic Stem Cell Transplantation Unit, Hematology Department, Institute of Hematology and Oncology, IDIBAPS, Hospital Clínic
[2] Department of Pharmacy, Pharmacy Service, Hospital Clínic
[3] Pharmacology and Toxicology Laboratory, Biochemistry and Molecular Genetics Department, Biomedical Diagnostic Center, IDIBAPS, CIBERehd, Hospital Clínic, Barcelona, Spain.
* Correspondence: **author:** Dr. Carmen Martínez, Hematology Department, Institute of Hematology and Oncology, Hospital Clínic, Villarroel 170, Barcelona, 08036. Spain. Phone: +34 93 227 54 28; Fax: +34 93 227 54 84; Email: cmarti@clinic.cat ORCID (Carmen Martínez): 0000-0002-8401-7306

SUPPLEMENTARY MATERIAL

Table S1. Impact of patient's and transplant's baseline characteristics on Tac IPV and TISS (univariate analysis).

Univariate analysis	Tac IPV >50th percentile Odds Ratio (95% CI)	P value	Tac TISS Odds Ratio (95% CI)	P value
**Increasing age**	0.98 (0.96-1.01)	0.25	1 (0.98-1.04)	0.49
**Sex**				
Female (vs. male)	1.55 (0.77-3.12)	0.22	1.94 (0.90-4.18)	0.09
**HCT Comorbidity Index** >2 (vs. $\leq$2)	0.87 (0.37-2)	0.74	1.16 (0.48-2.86)	0.75
**CMV risk** High (vs. Low, Intermediate)	0.91 (0.43-1.91)	0.79	0.71 (0.31-1.66)	0.44
**Pretransplant Renal Function** Creatinine, mg/dL	0.21 (0.036-1.24)	0.085	0.54 (0.084-3.53)	0.53
**Conditioning Regimen**				
RIC (vs. MAC)	1.46 (0.71-3.01)	0.29	1.26 (0.58-2.72)	0.56
**Donor Type**				
MMUD and haplo (vs. MSD and MUD)	0.64 (0.32-1.29)	0.21	0.71 (0.33-1.54)	0.39
**Graft source**				
Peripheral blood (vs. BM)	0.51 (0.04-5.74)	0.58	4.94 (0.43-56.24)	0.2
**Tacrolimus formulation at initiation**				
Oral (vs. IV)	6.44 (2.55-16.26)	<0.001	6.75 (1.93-23.68)	0.003

HR, hazard ratio; CI, confidence interval; aGVHD, acute graft versus host disease; RIC, reduced intensity conditioning; Tac, tacrolimus; IPV, intra patient variability; TISS, tacrolimus initial steady state.

Table S2. Impact of patient's and transplant's baseline characteristics on TISS (multivariate analysis).

Multivariate analysis	TISS Odds Ratio (95% CI)	P value
**Sex**		
Female (vs. male)	2.10 (0.94-4.70)	0.072
**Tacrolimus formulation at initiation**		
**Oral (vs. IV)**	7.09 (1.99-25.16)	0.002

Because no other baseline characteristics were associated with an increased probability of high Tac IPV (>50th percentile), multivariate analysis was not performed.

The above analysis confirmed the results showing the association between route of Tac administration and the risk of high Tac IPV (>50th percentile) or TISS. A non-significant correlation between female sex and IPSS was documented, with no other association between any of the other baseline characteristics.

Table S3a. Multivariate analysis of risk factors for aGVHD using 25th percentile cut-off.

		Grade II-IV aGVHD HR (95% CI)	P	Grade III-IV aGVHD HR (95% CI)	P
**Patient Age**	Continuous variable	1.04 (1.00-1.07)	0.015	1.12 (1.05-1.2)	0.001
**Conditioning Regimen**	Myeloablative (vs. RIC)	5.37 (2.23-12.95)	<0.001	14.55 (4.04-52.39)	<0.001
**Donor Type**	Mismatch (vs. match)	0.61 (0.29-1.28)	0.19	0.8 (0.21-3.03)	0.74
**Tac IPV**	>25th percentile (vs. ≤25th)	0.57 (0.18-1.82)	0.35	0.32 (0.032-3.17)	0.33
**Tac TISS**	≥5 ng/mL	1.78 (0.8-3.99)	0.16	1.11 (0.31-4)	0.88

HR, hazard ratio; CI, confidence interval; aGVHD, acute graft versus host disease; RIC, reduced intensity conditioning; Tac, tacrolimus; IPV, intra patient variability; TISS, tacrolimus initial steady state.

Table S3b. Multivariate analysis of risk factors for aGVHD using 75th percentile cut-off.

		Grade II-IV aGVHD HR (95% CI)	P	Grade III-IV aGVHD HR (95% CI)	P
**Patient Age**	Continuous variable	1.03 (0.99-1.06)	0.06	1.11 (1.03-1.19)	0.007
**Conditioning Regimen**	Myeloablative (vs. RIC)	4.64 (1.82-11.78)	0.001	11.04 (2.27-53.73)	0.003
**Donor Type**	Mismatch (vs. match)	0.64 (0.3-1.36)	0.25	0.79 (0.19-3.35)	0.75
**Tac IPV**	>75th percentile (vs. ≤75th)	2.38 (1.08-5.27)	0.03	3.7 (0.63-21.72)	0.15
**Tac TISS**	≥5 ng/mL	1.56 (0.65-3.72)	0.32	0.85 (0.18-3.99)	0.84

HR, hazard ratio; CI, confidence interval; aGVHD, acute graft versus host disease; RIC, reduced intensity conditioning; Tac, tacrolimus; IPV, intra patient variability; TISS, tacrolimus initial steady state.
